# Supplementary material for: Discrimination reveals reconstructability of multiplex networks from partial observations
Source: Commun Phys. 2022 Jun 27;5(1):163. doi: 10.1038/s42005-022-00928-w (PMC9243819; doi:10.1038/s42005-022-00928-w)
Supplement: Supplementary file 2 — Supplementary Information [file 42005_2022_928_MOESM2_ESM.pdf]

Supplementary Information for

Discrimination reveals reconstructability of multiplex networks from aggregate and partial observations

Mincheng Wu, Jiming Chen, Shibo He, Youxian Sun, Shlomo Havlin, Jianxi Gao

Supplementary Contents

Supplementary Figure 1 · · · · · 3
Supplementary Figure 2 · · · · · 4
Supplementary Figure 3 · · · · · 5
Supplementary Figure 4 · · · · · 6
Supplementary Figure 5 · · · · · 7
Supplementary Figure 6 · · · · · 8
Supplementary Figure 7 · · · · · 9
Supplementary Figure 8 · · · · · 10
Supplementary Figure 9 · · · · · 11
Supplementary Figure 10 · · · · · 12
Supplementary Figure 11 · · · · · 13
Supplementary Figure 12 · · · · · 14
Supplementary Figure 13 · · · · · 15
Supplementary Figure 14 · · · · · 16
Supplementary Figure 15 · · · · · 17
Supplementary Table 1 · · · · · 19
Supplementary Table 2 · · · · · 25
Supplementary Note 1 · · · · · 27
Supplementary Note 2 · · · · · 29

Supplementary Note 3 · · · · · · · · · · 34
Supplementary Note 4 · · · · · · · · · · 34
Supplementary Note 5 · · · · · · · · · · 35
Supplementary Note 6 · · · · · · · · · · 36
Supplementary Note 7 · · · · · · · · · · 37
Supplementary References · · · · · · · · · · 39

Supplementary Figures

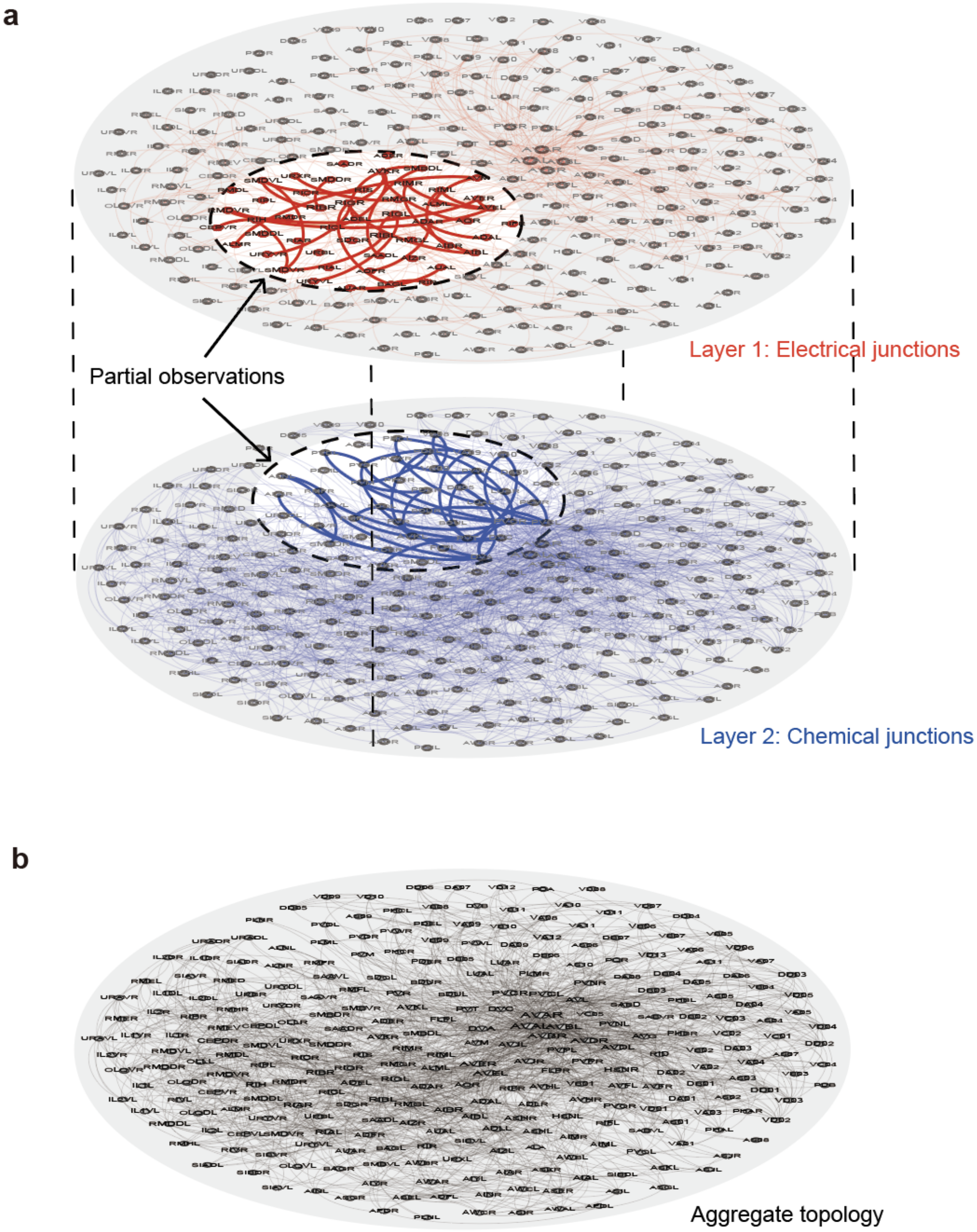

Supplementary Figure 1: **The multiplex network composed of C. elegans neuronal connectome. a** The multiplex network composed of two layers, indicating electrical junctions and chemical junctions, respectively. The highlighted edges in the subgraphs are the partial observations denoted by Γ. **b** The aggregate topology of the C. elegans multiplex connectome shown in **a**, which is a monoplex network aggregated by the OR mechanism.

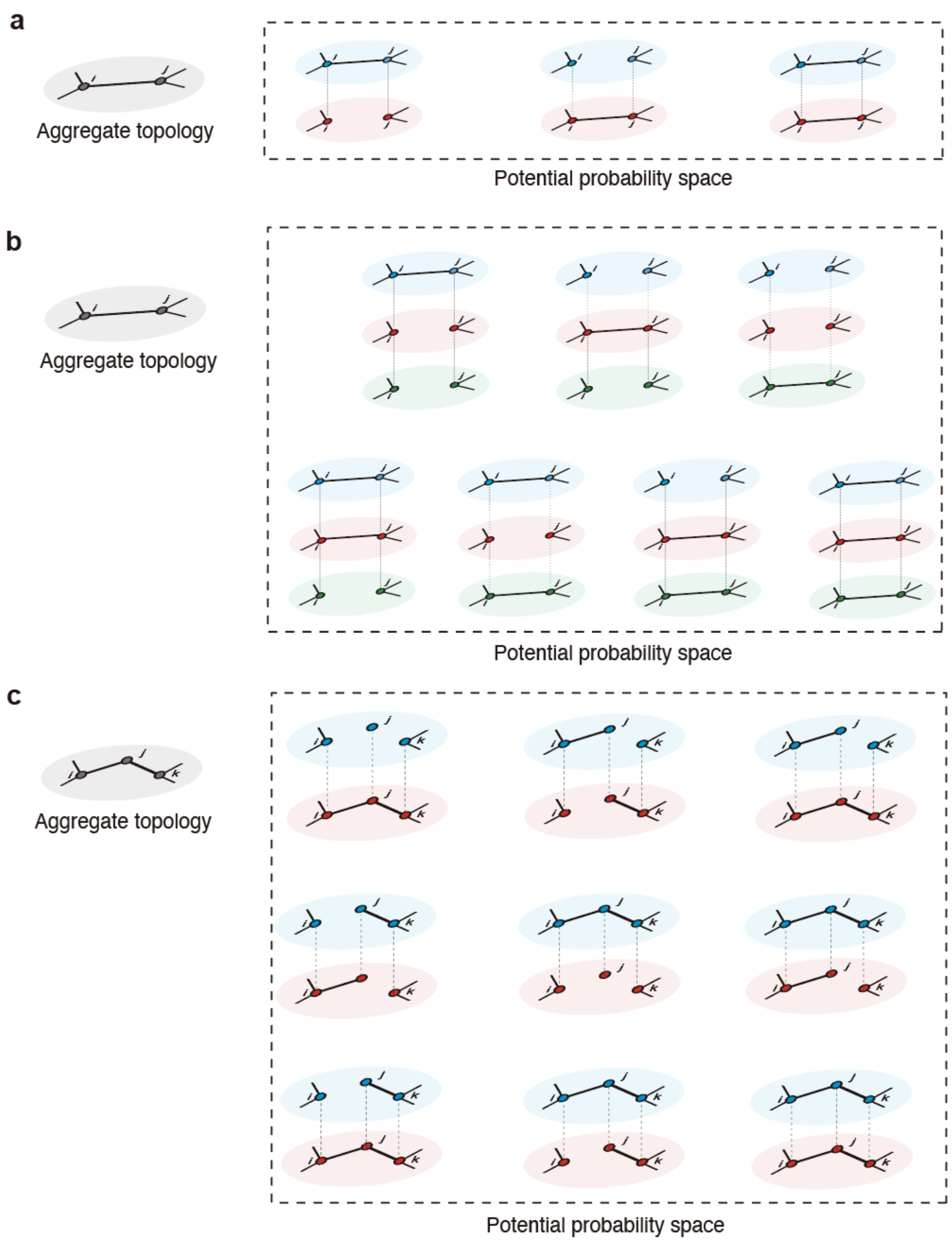

Supplementary Figure 2: **The exponential-growth probability space.** **a** When an individual link between node i and node j is observed in the aggregate topology, it might exist only in layer 1, only in layer 2, or in both layers, composing a three-events probability space for the multiplex structure. **b** The probability space of a three-layer ($L = 3$) potential multiplex structure corresponding to an individual observed link, which is composed of seven ($2^L - 1$) events leading to an exponential-growth. **c** Once two links between nodes i, j and nodes j, k are observed in the aggregate topology ($|A^{\mathcal{O}}| = 2$), the number of potential events grows to nine ($3^{|A^{\mathcal{O}}|}$), resulting an exponential-growth as well.

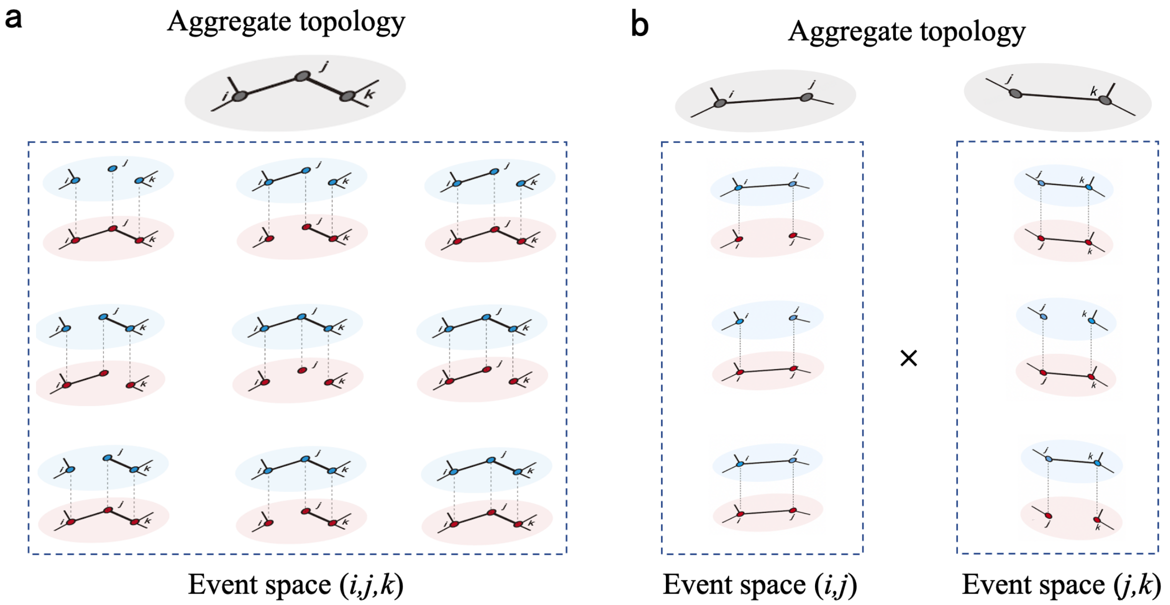

Supplementary Figure 3: **A schematic illustration for the complexity dropping.** **a** shows an example of the aggregated network consisting of three nodes (i, j, k) and two edges between them. For a two-layer multiplex network, there are $9(3^2)$ events in the event space, leading to an exponentially computational complexity, i.e., $O(3^m)$. **b** shows the independent assumption for the edge between i and j and the edge between j and k. In this case, the event space (i, j, k) can be presented as the product of two independent event spaces, each of which has 3 events. Therefore, the computational complexity has a linear growth, i.e., $O(3 \cdot m)$.

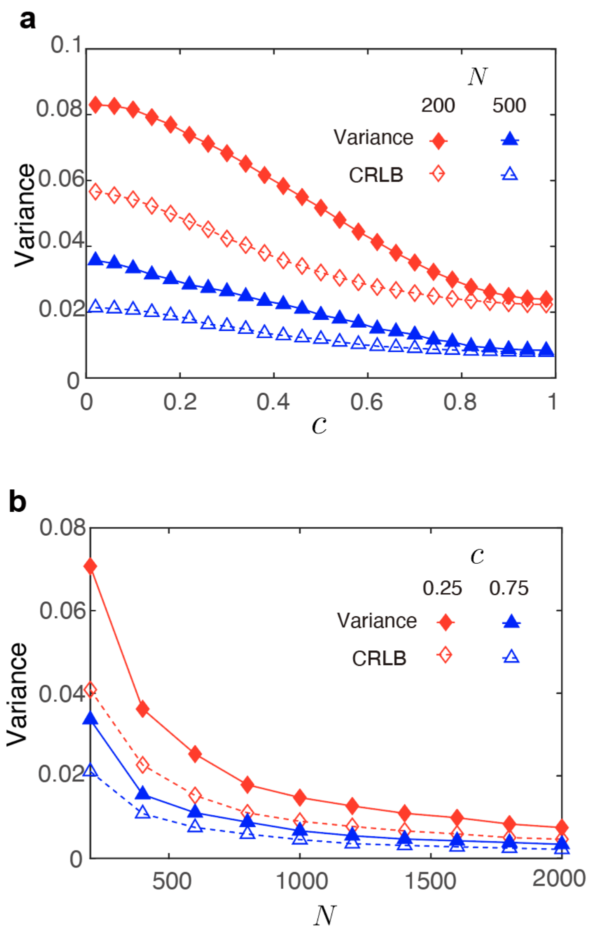

Supplementary Figure 4: **The analysis for the variance of the estimator.** The mean variances of estimated parameters and the corresponding Cramer-Rao lower bounds are shown in **a** and **b**, ranging c and N, respectively. These results are obtained from synthetic networks by repeating 1,000 times.

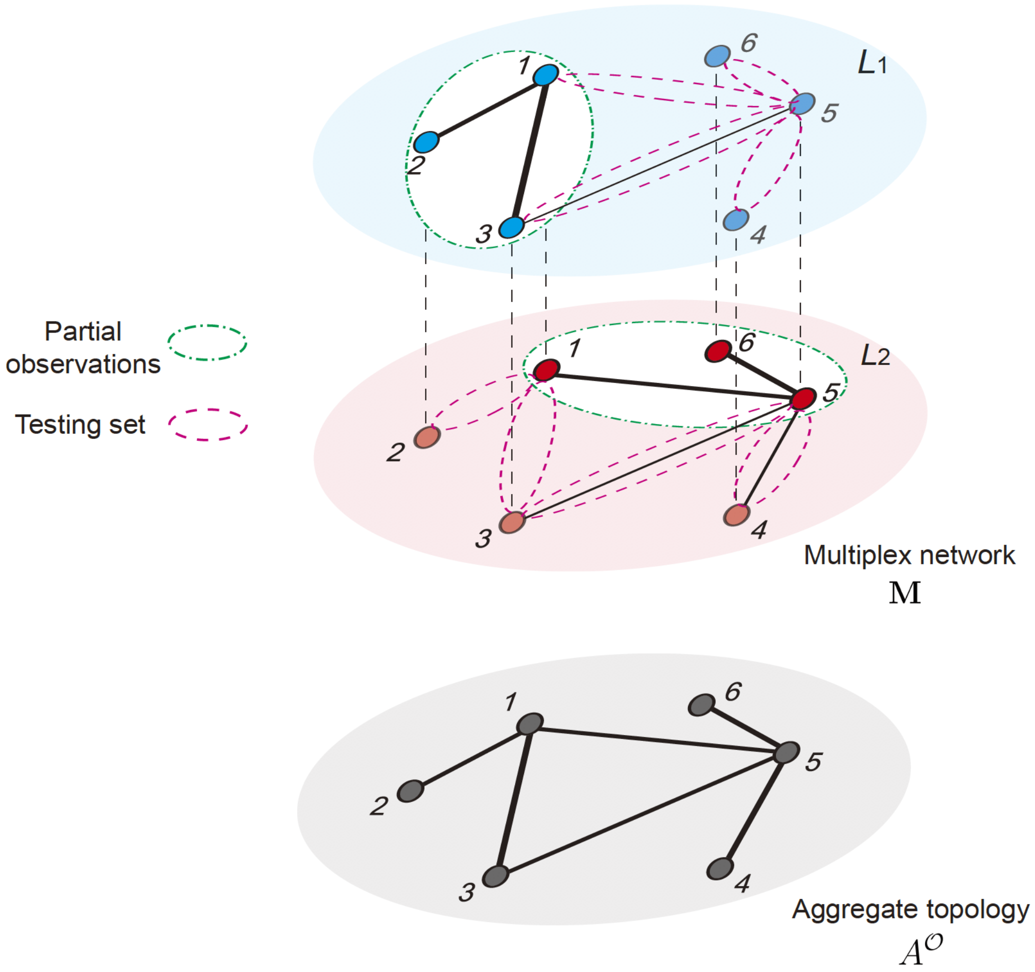

Supplementary Figure 5: **The illustration of observations and testing set.** The potential edges surrounded by red circles are the testing set E^T consisting of potential edges except partial observations surrounded by green circles, i.e., $E^T = \{M_{ij}^{\alpha} \notin \Gamma | A_{ij}^{\mathcal{O}} = 1\}$.

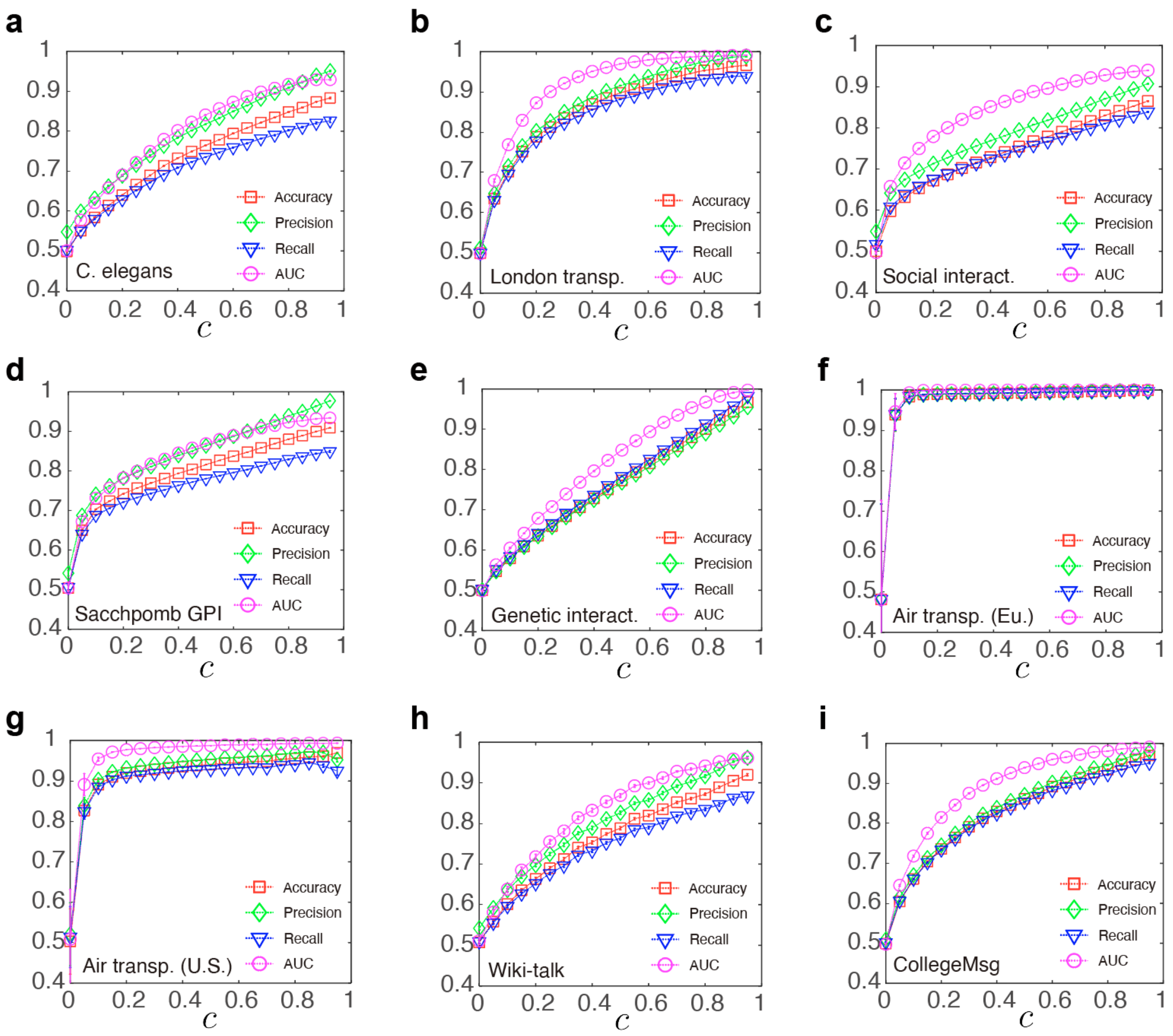

Supplementary Figure 6: **Four evaluations for reconstruction.** The accuracy, precision, recall and AUC are tested against c (fraction of partial observations). These results are obtained by repeating 1,000 times from nine real-world networks: **a**, C. elegans connectome; **b**, London transportation; **c**, Social interaction at SFHH; **d**, Sacchpomb GPI; **e**, Genetic interaction; **f**, air transportation in Europe; **g**, air transportation in the U.S.; **h**, Wiki-talk network; **i**, CollegeMsg network.

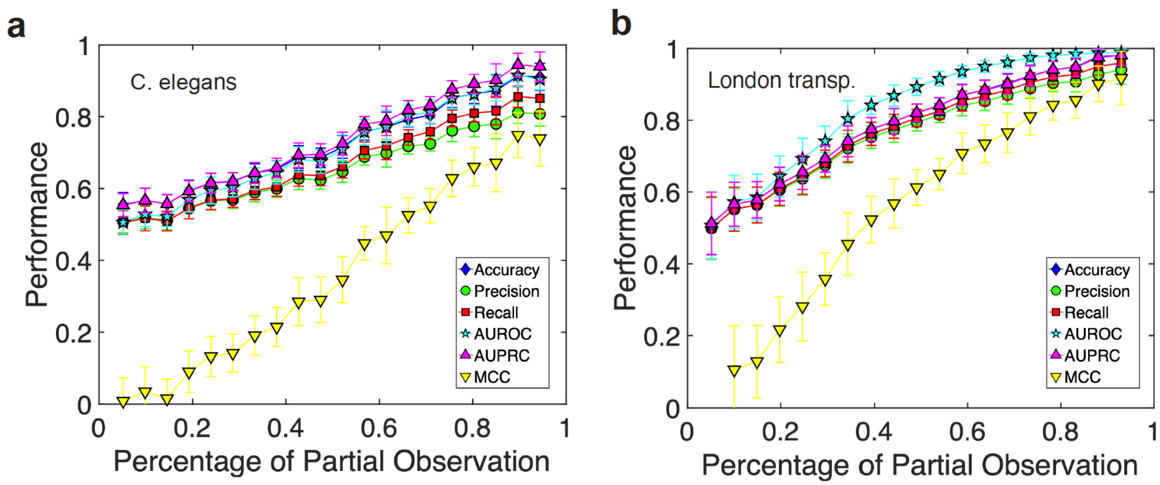

Supplementary Figure 7: **More metrics for evaluations.** The AUPRC (area under the precision-recall curve) is also performed well in the two empirical networks. Notice that the MCC (Matthews correlation coefficient) ranges in the interval $[-1, +1]$, with extreme values -1 and $+1$ reached in case of perfect misclassification and perfect classification, respectively[1]. The results of AUPRC and MCC are consistence with the other metrics considered from two real-world networks: **a** C. elegans connectome; **b** London transportation network.

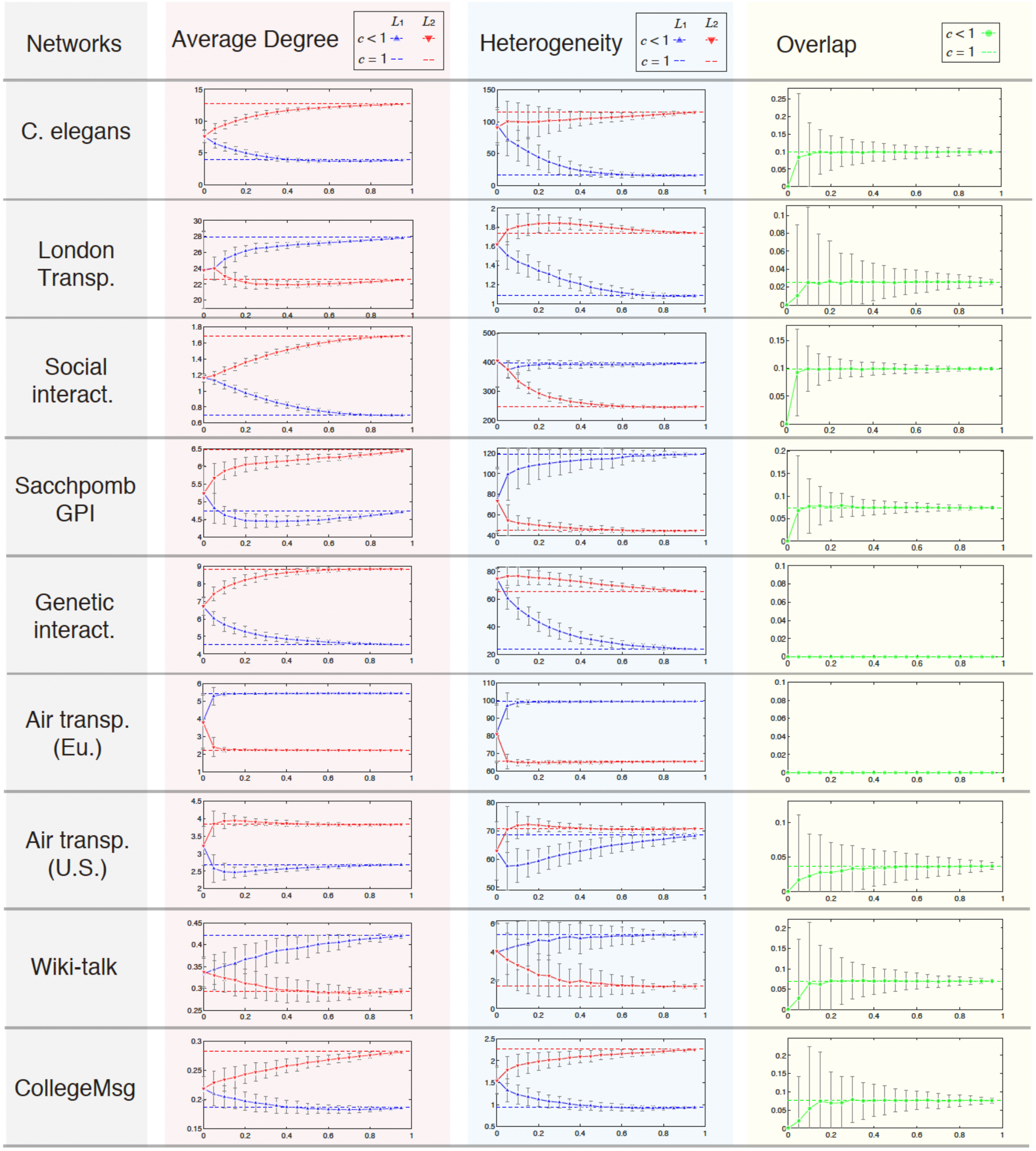

Supplementary Figure 8: **The mesoscale structure revealed in reconstructed multiplex networks.** The average degree, the heterogeneity of each layer, and the overlap of edges in the reconstructed network for nine real-world networks with c increasing.

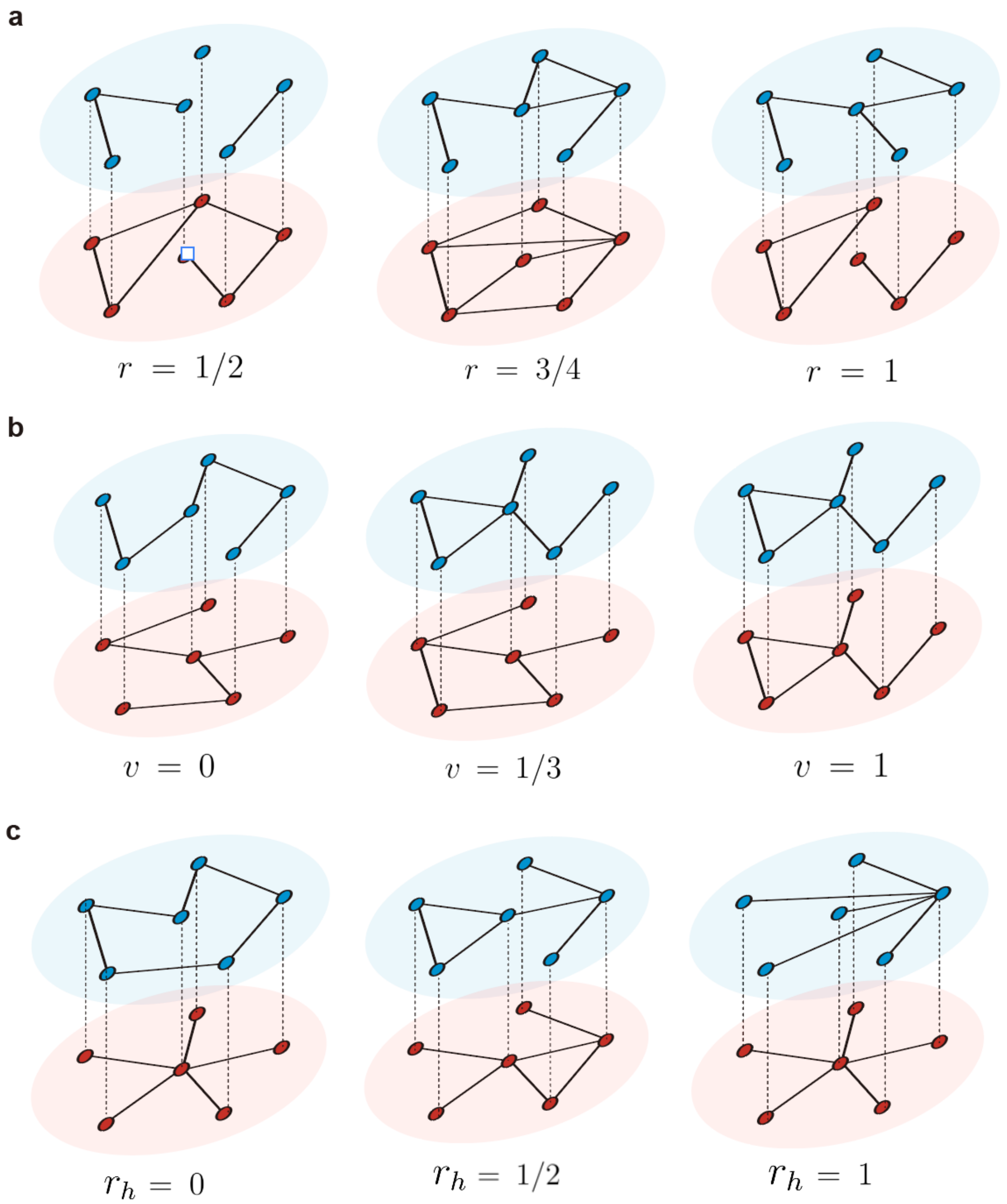

Supplementary Figure 9: **Toy examples for various multiplex network characteristics.** We consider three main multiplex network characteristics for illustration. Three toy examples with different characteristics are presented for **a**, $r = 1/2$,$3/4$, and 1; **b**, $v = 0$,$1/3$ and 1; **c**, $r_h = 0$, $1/2$, and 1.

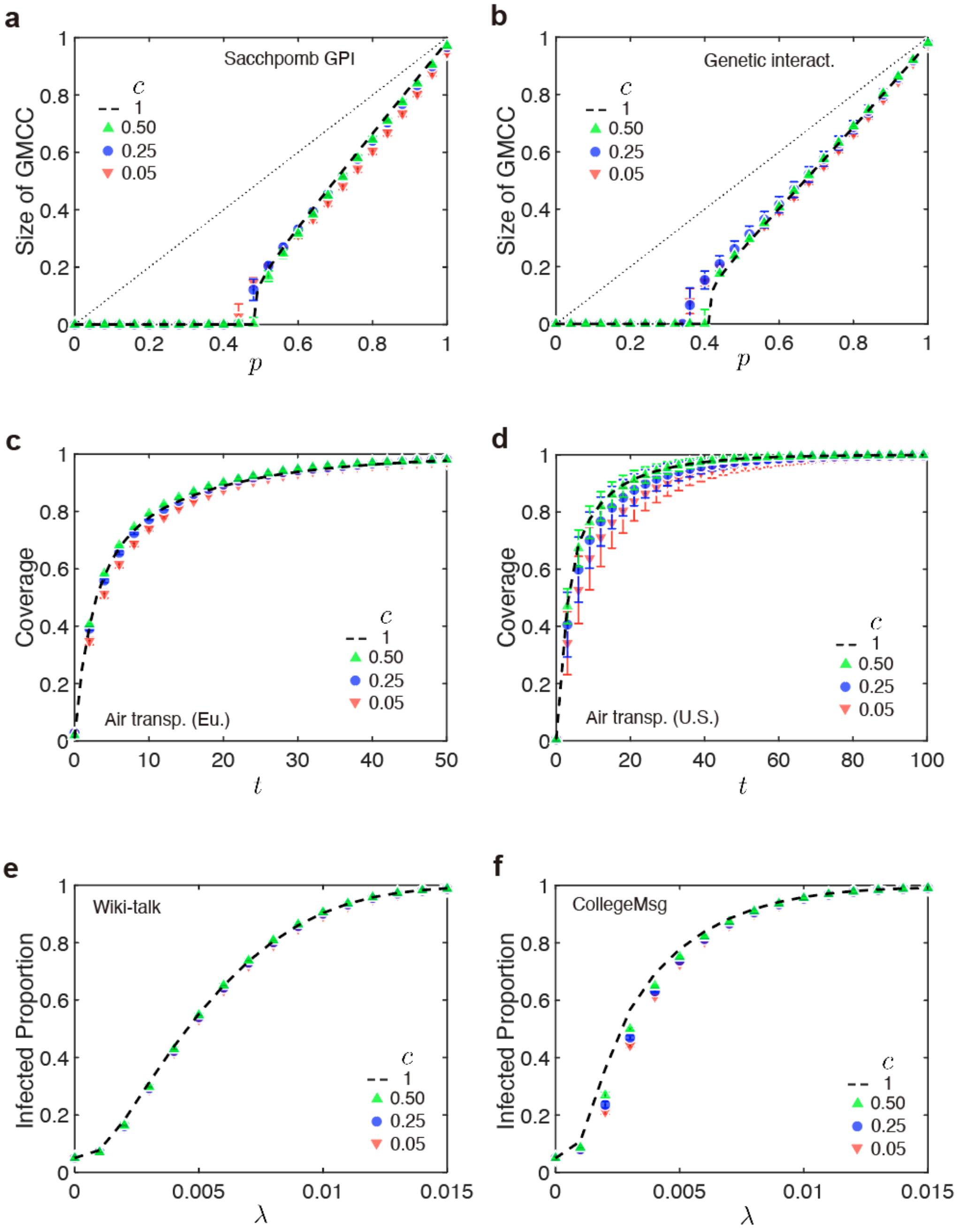

Supplementary Figure 10: **The dynamics taking place on reconstructed networks.** The percolation processes of the reconstructed multiplex networks (c = 0.05, 0.25, 0.5) and the real multiplex networks (c = 1) for **a**, Sacchpomb genetic-protein interactions and **b**, Yeast genetic interactions. The random walk process taking place on the reconstructed multiplex networks (c = 0.05, 0.25, 0.5) and real multiplex networks (c = 1) for **c**, air transportation networks of Europe and **d**, air transportation networks of the United States. The spreading process on the reconstructed temporal networks (c = 0.05, 0.25, 0.5) and real temporal networks (c = 1) for **e**, the Wiki-talk network and **f**, the CollegeMsg network.

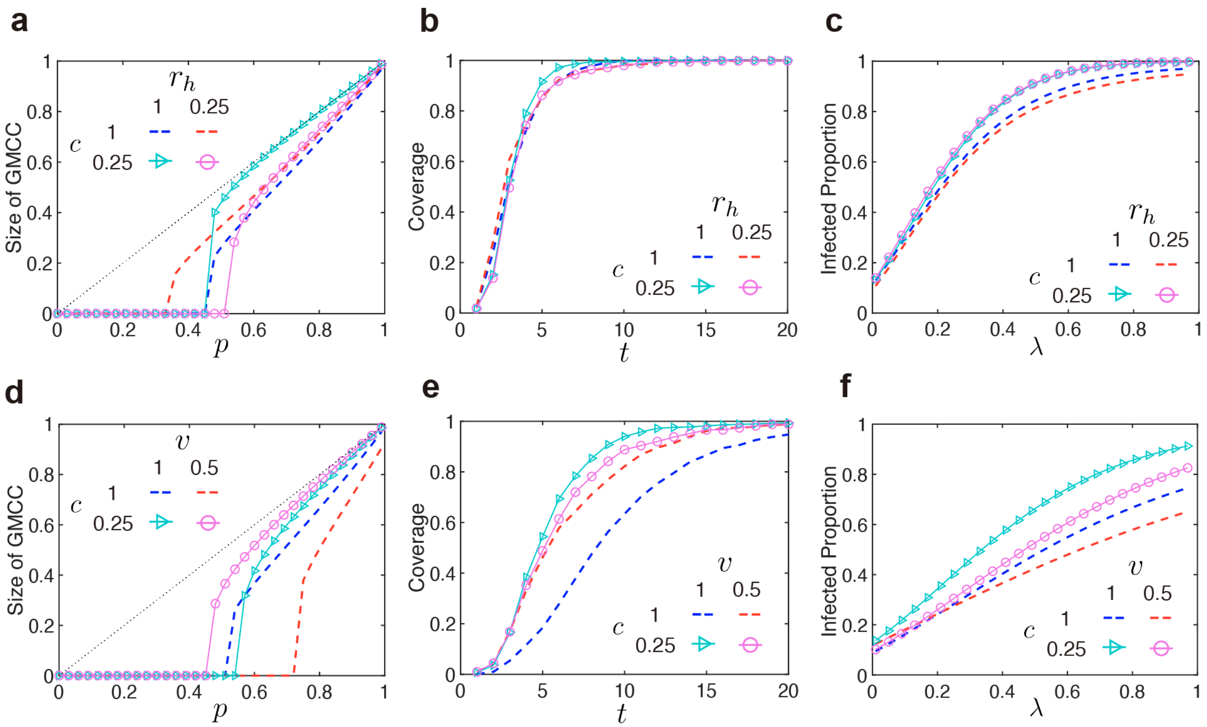

Supplementary Figure 11: **The dynamic processes influenced by various multiplex network characteristics.** Synthetic networks shows the impact of different r_h on the dynamic processes: **a**, percolation process; **b**, random walk process and **c**, spreading process. Synthetic networks shows the impact of different v on the dynamic processes: **d**, percolation process; **e**, random walk process and **f**, spreading process.

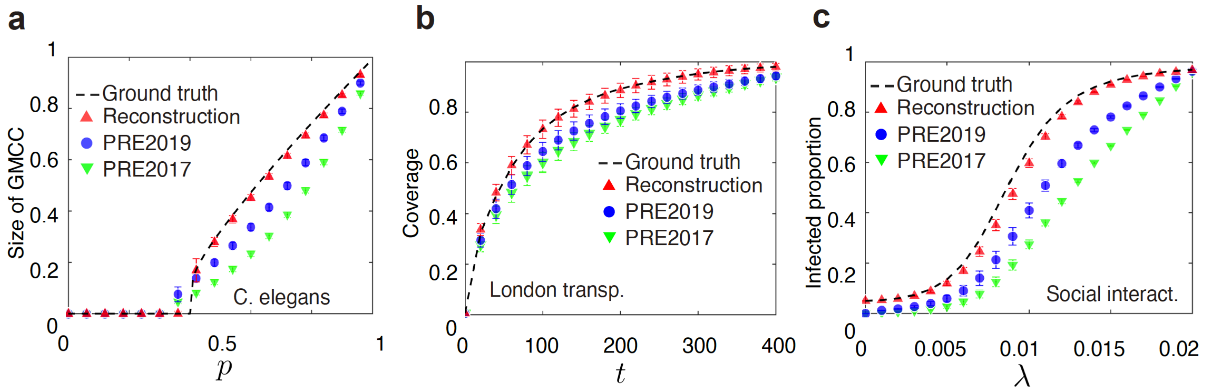

Supplementary Figure 12: **The performance of dynamic prediction with different methods.** **a** The percolation processes of the reconstructed multiplex network ($c = 0.5$) for C. elegans multiplex connectome. The x-axis denotes the occupied probability p and the y-axis denotes the size of GMCC when nodes are randomly removed with probability $1 - p$ in one layer. **b** A random walk process taking place on the reconstructed multiplex network and real multiplex network for London transportation network. The x-axis denotes time t and the y-axis denotes coverage (the fraction of nodes that have been visited before a certain time) of n walkers starting from a set of random chosen nodes. **c** The spreading process on the reconstructed temporal network and real temporal network for the social interactions at the SFHH. The x-axis denotes the infection rate and the y-axis denotes the infected fraction.

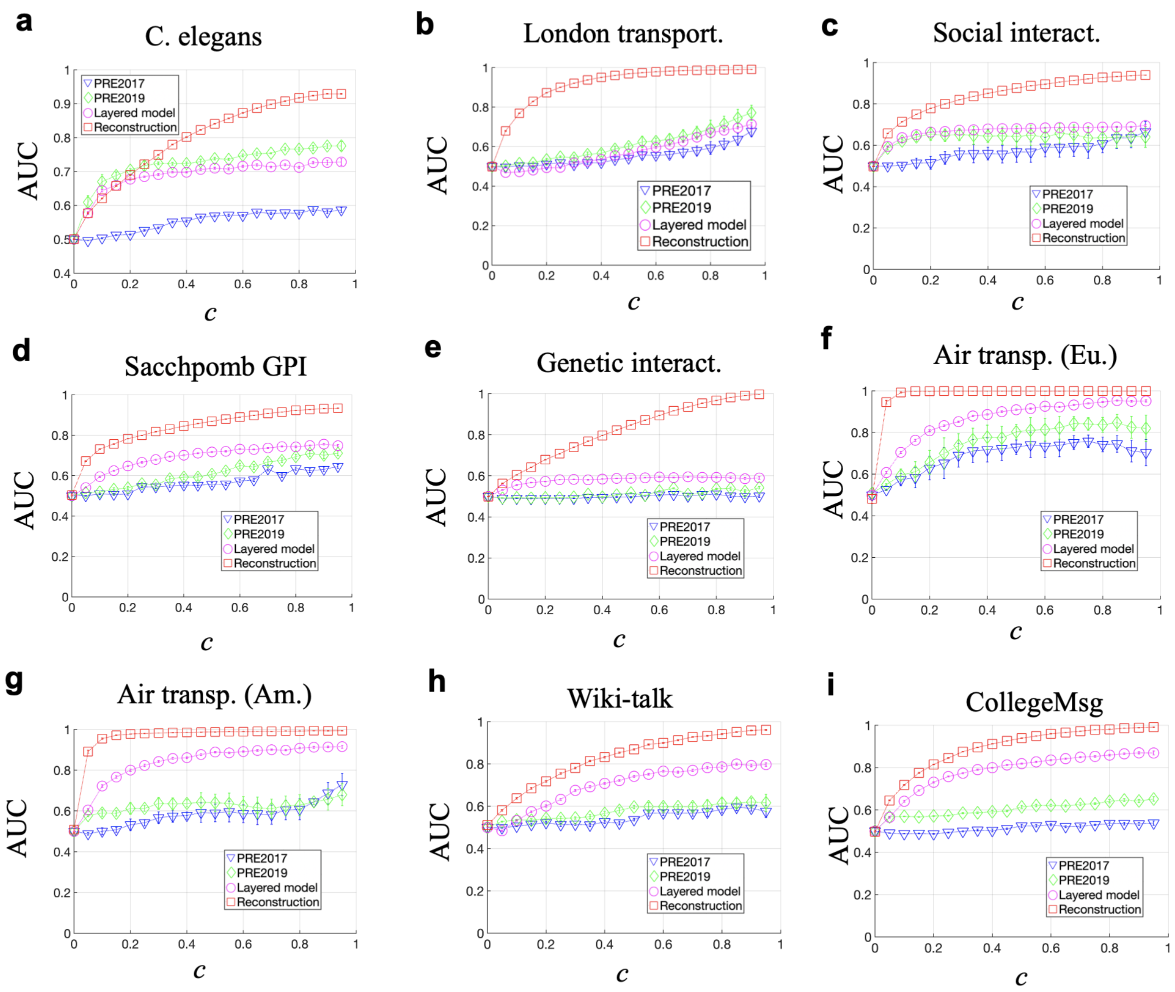

Supplementary Figure 13: **The performance compared to related works**. We compared the performance of our reconstruction framework with three baselines. The first relevant work is that of De Bacco et al. PRE2017[2], and the second is at of Tarres-Deulofeu et al. PRE2019[3]. The third baseline is calculated by single-layer link prediction method layer by layer in the multiplex network (Layered model). We compare the AUC obtained these four methods for the nine real-world multiplex networks, including biological networks, transportation networks, and social networks, finding that our method is the most effective.

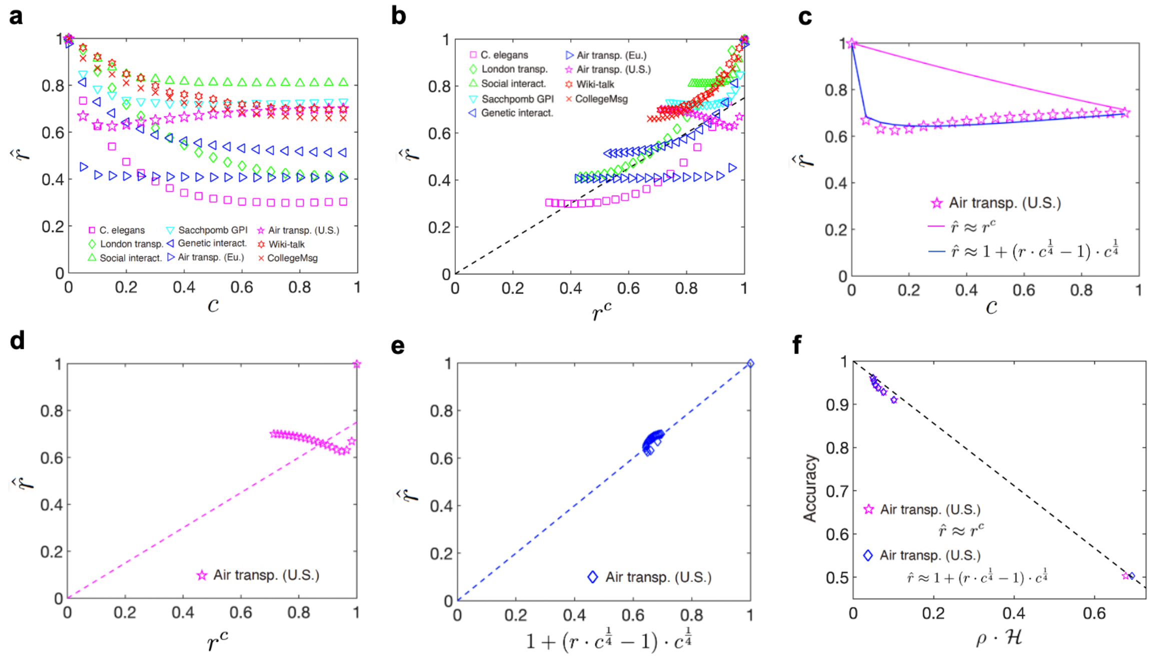

Supplementary Figure 14: **The performance of the approximation for** $\hat{r}$. **a** The empirical results for the approximated ratio of average degree $\hat{r}$ ranging c from 0 to 1 for nine real-world networks. **b** We approximated $\hat{r}$ by $\hat{r} \approx r^c$, and the Pearson coefficient between them reaches 0.89 in all empirical networks. **c** The star markers indicate the empirical results for $\hat{r}$ in the considered network. The pink curve indicates the approximation for $\hat{r}(r, c)$ by $\hat{r} \approx r^c$, and the blue curve indicates the approximation for $\hat{r}(r, c)$ by $\hat{r} \approx 1 + (r \cdot c^{\frac{1}{4}} - 1) \cdot c^{\frac{1}{4}}$. **d** We approximate $\hat{r}(r, c)$ by $\hat{r} \approx r^c$ for the considered network, and the Pearson coefficient between them reaches 0.79. **e** We approximate $\hat{r}(r, c)$ by $\hat{r} \approx 1 + (r \cdot c^{\frac{1}{4}} - 1) \cdot c^{\frac{1}{4}}$ for the considered network, and the Pearson coefficient between them reaches 0.98. **f** The two different approximations show the Pearson correlation between accuracy of reconstruction and the discrimination indicator are both 0.97.

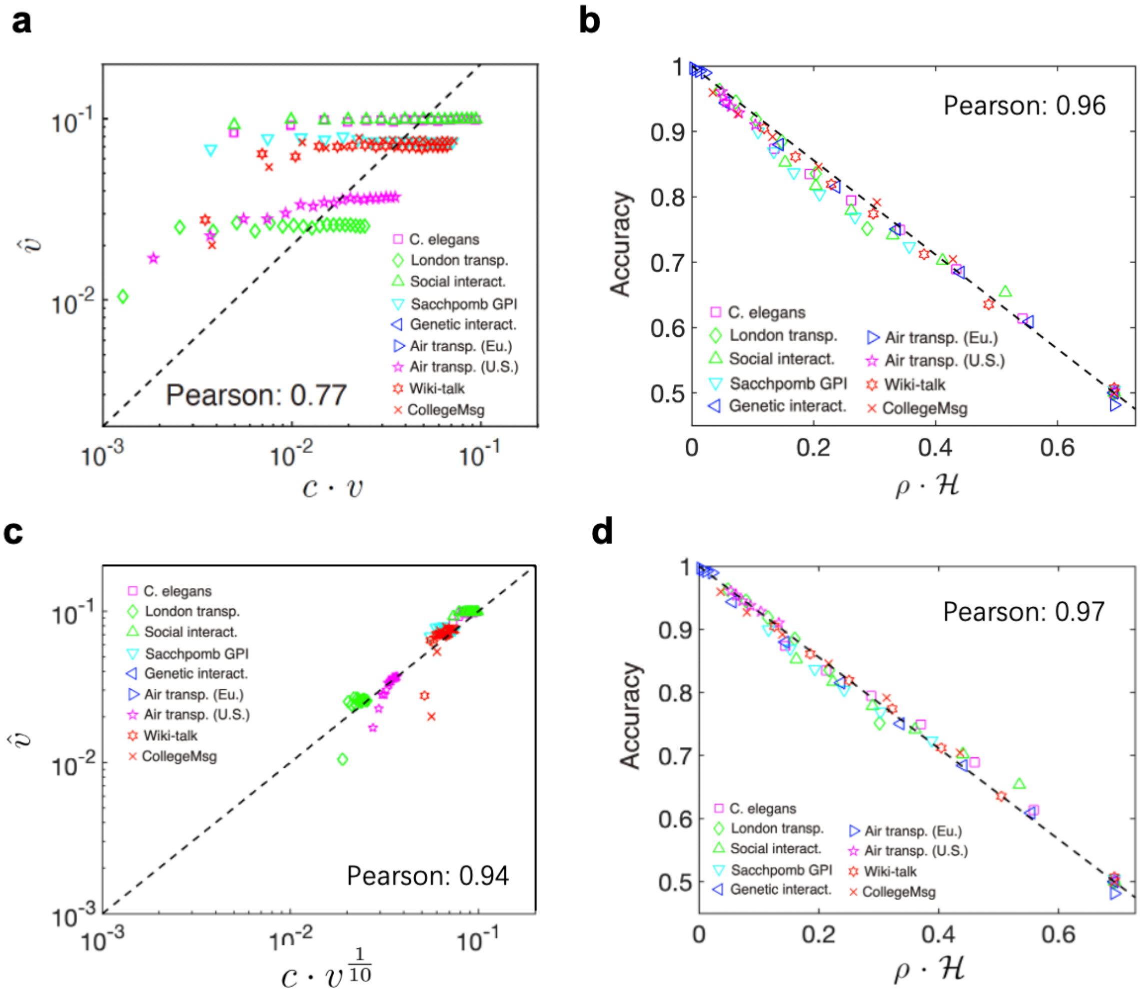

Supplementary Figure 15: **The performance of the approximation for** $\hat{v}$. **a** We approximate $\hat{v}(v, c)$ by $\hat{v} \approx c \cdot v$, and the Pearson coefficient between them reaches 0.77 in all empirical networks. **b** The relationship between accuracy of reconstruction and the discrimination indicator for nine real-world networks is displayed, where the Pearson correlation is 0.96. **c** We approximate $\hat{v}(v, c)$ by $\hat{v} \approx c \cdot v^{\frac{1}{10}}$, and the Pearson coefficient between them reaches 0.94 in all empirical networks. **d** The relationship between accuracy of reconstruction and the discrimination indicator for nine real-world networks is displayed, where the Pearson correlation is 0.97.

Supplementary Tables

Network	Layers	N	$\|E\|$	$\langle k \rangle$	h	GCC
C. elegans connectome [4,5]	Electric	213	415	3.90	16.32	213
	Chemical	213	1353	12.70	115.83	213
London transp. [6]	Overground	369	129	0.70	1.09	126
	Underground	369	312	1.69	1.074	271
Social interact. at the SFHH [7]	June 4	320	4464	27.90	396.37	320
	June 5	320	3622	22.64	247.39	320
Sacchpomb GPI network [8,9]	Physical association	530	1254	4.73	119.01	530
	Suppressive interaction	530	1715	6.47	44.63	530
Yeast genetic interact. [10]	Positive	506	1145	4.53	23.77	506
	Negative	506	2232	8.82	65.47	506
Air transp. (Eu.) [11]	Ryanair	220	601	5.46	99.57	128
	Lufthansa	220	244	2.22	65.36	106
	EasyJet	220	307	2.79	46.17	99

continued on next page

continued from previous page

Network	Layers	N	$\|E\|$	$\langle k \rangle$	h	GCC
Air transp. (U.S.)	SkyWest	214	288	2.69	68.54	144
	Southwest	214	411	3.84	70.94	64
	American Eagle	214	191	1.79	40.60	113
	American Airlines	214	214	2.00	45.30	78
Social interact. (22 layers) [7]	Hour 1	403	154	0.76	6.69	60
	Hour 2	403	144	0.71	5.54	37
	Hour 3	403	1522	7.55	83.62	255
	Hour 4	403	561	2.78	17.59	214
	Hour 5	403	550	2.73	15.79	212
	Hour 6	403	661	3.28	23.91	211
	Hour 7	403	558	2.77	19.01	214
	Hour 8	403	1841	9.14	78.87	337
	Hour 9	403	861	4.27	27.69	276
	Hour 10	403	554	2.75	10.56	250

continued on next page

continued from previous page

Network	Layers	N	$\|E\|$	$\langle k \rangle$	h	GCC
	Hour 11	403	136	0.67	3.06	66
	Hour 12	403	20	0.10	0.33	13
	Hour 13	403	31	0.15	0.55	24
	Hour 14	403	72	0.36	1.17	28
	Hour 15	403	193	0.96	3.81	98
	Hour 16	403	306	1.52	7.34	119
	Hour 17	403	3058	15.18	203.89	333
	Hour 18	403	482	2.39	12.64	182
	Hour 19	403	494	2.45	11.83	220
	Hour 20	403	459	2.28	11.40	195
	Hour 21	403	287	1.42	6.88	121
	Hour 22	403	35	0.17	0.81	20
Wiki-talk network [12,13]	Week 1	1115	235	0.42	4.97	145
	Week 2	1115	164	0.29	1.49	117

continued on next page

continued from previous page

Network	Layers	N	$\|E\|$	$\langle k\rangle$	h	GCC
	Week 3	1115	186	0.33	3.00	120
	Week 4	1115	161	0.29	1.71	102
	Week 5	1115	249	0.45	3.20	138
	Week 6	1115	190	0.34	1.93	119
	Week 7	1115	301	0.54	6.96	167
	Week 8	1115	258	0.46	4.85	168
	Week 9	1115	335	0.60	7.35	194
	Week 10	1115	362	0.65	6.26	213
	Week 11	1115	334	0.60	5.60	187
	Week 12	1115	414	0.74	7.82	235
	Week 13	1115	428	0.77	8.16	223
	Week 14	1115	439	0.79	10.55	274
CollegeMsg network [14]	Day 1	1209	113	0.19	0.61	78
	Day 2	1209	171	0.28	1.46	115

continued on next page

continued from previous page

Network	Layers	N	$\|E\|$	$\langle k \rangle$	h	GCC
	Day 3	1209	164	0.27	1.06	107
	Day 4	1209	145	0.24	0.66	101
	Day 5	1209	268	0.44	1.80	149
	Day 6	1209	250	0.41	1.32	156
	Day 7	1209	327	0.54	1.99	182
	Day 8	1209	426	0.70	3.97	222
	Day 9	1209	333	0.55	2.00	201
	Day 10	1209	275	0.45	1.64	183
	Day 11	1209	406	0.67	2.69	231
	Day 12	1209	630	1.04	5.94	325
	Day 13	1209	583	0.96	4.01	310
	Day 14	1209	622	1.03	4.74	330
	Day 15	1209	700	1.16	5.41	356
	Day 16	1209	676	1.12	17.15	436

continued on next page

continued from previous page

Network	Layers	N	$\|E\|$	$\langle k \rangle$	h	GCC
	Day 17	1209	399	0.66	2.46	263
	Day 18	1209	576	0.95	4.31	333
	Day 19	1209	233	0.39	1.14	146
	Day 20	1209	566	0.94	3.18	359
	Day 21	1209	508	0.84	2.73	341
	Day 22	1209	117	0.19	0.29	77
	Day 23	1209	439	0.73	3.87	307
	Day 24	1209	326	0.54	1.65	243
	Day 25	1209	468	0.77	2.82	305
	Day 26	1209	613	1.01	7.63	392
	Day 27	1209	530	0.88	2.64	342
	Day 28	1209	579	0.96	3.50	351

Supplementary Table 1: **Datasets overview.** We list all multiplex networks and their properties including the number of nodes (N), number of edges ($|E|$), average degree ($\langle k \rangle$), variance of degree distribution (h) and giant connected component (GCC).

Network	Layers	$\cos\left\langle \vec{d}^1, \vec{d}^2 \right\rangle$	$s_{(\mathbf{M})}$
C. elegans	Electric	0.85	0.80
	Chemical		
London transp.	Overground	0.13	0.28
	Underground		
Social interact.	June 4	0.81	0.70
	June 5		
Sacchpomb GPI network	Physical association	0.31	0.37
	Suppressive interaction		
Yeast genetic interact.	Positive	0.82	0.85
	Negative		
Air transp. (Eu.)	Ryanair	0.04	0.02
	Lufthansa		
Air transp. (U.S.)	SkyWest	0.24	0.08
	Southwest		

continued on next page

continued from previous page

Network	Layers	$\cos\left\langle \vec{d^1}, \vec{d^2} \right\rangle$	$s_{(\mathbf{M})}$
Wiki-talk network	Week 1	0.62	0.63
	Week 2		
CollegeMsg network	Day 1	0.56	0.52
	Day 2		

Supplementary Table 2: **Cosine similarity and s of each multiplex network.** We list all datasets tested in the analysis of entropy, and their properties including cosine similarity $\cos\left\langle \vec{d^1}, \vec{d^2} \right\rangle$ and $s_{(\mathbf{M})}$.

Supplementary Note 1: Aggregate mechanisms

There are many aggregate mechanisms that can map a multiplex network $\mathbf{M}$ to a monoplex network $A^{\mathcal{O}}$. Here we list three common cases. Observing the aggregation with logical relationship "OR" is the most common mechanism in real life. We adopt this aggregate mechanism in this article for illustration, and denote the mapping with relationship "OR" by φ_{OR}. Then we have

$$A^{\mathcal{O}} = \varphi_{\text{OR}}(\mathbf{M}) = \mathbf{1}_{N\times N} - \prod_{\alpha=1}^{L}(\mathbf{1}_{N\times N} - M^{\alpha}),$$

where $\mathbf{1}_{N\times N} \in \mathbb{R}^{N\times N}$ is the matrix with all elements equaling to one, and

$$A^{\mathcal{O}}_{ij} = \varphi_{\text{OR}}(\mathbf{M}_{ij}) = 1 - \prod_{\alpha=1}^{L}(1 - M^{\alpha}_{ij}).$$

The "OR" aggregate mechanism maps an unweighted multiplex network to an unweighted monoplex network. For example, assuming that the α-th layer M^{α} is an undirected, unweighted network generated by the ER network model with parameter θ^{α}, the distribution of a link $A^{\mathcal{O}}_{ij}$ in the aggregate network $A^{\mathcal{O}}$ with "OR" is submitted to a Bernoulli distribution

$$P(A^{\mathcal{O}}_{ij} = k) = \begin{cases} 1 - \prod_{\alpha}(1-\theta^{\alpha}), & \text{if } k = 1 \\ \prod_{\alpha}(1-\theta^{\alpha}), & \text{if } k = 0 \end{cases}.$$

Then, the joint distribution of observing the whole aggregate network is

$$P(A^{\mathcal{O}}|\Theta) = \prod_{i<j}\left[1 - \prod_{\alpha}(1-\theta^{\alpha})\right]^{A^{\mathcal{O}}_{ij}} \cdot \left[\prod_{\alpha}(1-\theta^{\alpha})\right]^{1-A^{\mathcal{O}}_{ij}}.$$

The second mechanism obtains the aggregate network by simple aggregation with summation, i.e.,

$$A^{\mathcal{O}} = \varphi_{\text{SUM}}(\mathbf{M}) = \sum_{\alpha=1}^{L} M^{\alpha},$$

and

$$A^{\mathcal{O}}_{ij} = \varphi_{\text{SUM}}(\mathbf{M}_{ij}) = \sum_{\alpha=1}^{L} M^{\alpha}_{ij}.$$

This aggregation can map an unweighted multiplex network to a weighted monoplex network. Specifically, if M^α is an unweighted multiplex network, i.e., $M_{ij}^\alpha \in \{0, 1\}$, the aggregate network may not be unweighted network any longer, since $A_{ij}^{\mathcal{O}} \in \{0, 1, 2, \cdots, L\}$. For example, suppose that the α-th layer M^α is an undirected, unweighted network generated by the ER network model with parameter θ, i.e.,

$$P(M_{ij}^\alpha = k) = \begin{cases} \theta \ , & \text{if } k = 1 \\ 1-\theta, & \text{if } k = 0 \end{cases}.$$

The distribution of an individual link $A_{ij}^{\mathcal{O}}$ in the aggregate network $A^{\mathcal{O}}$ with the "SUM" mechanism is submitted to a multinomial distribution

$$P(A_{ij}^{\mathcal{O}} = k) = \binom{L}{k} \cdot \theta^k (1-\theta)^{L-k}, \ k = 0, 1, 2, \cdots, L.$$

Then, we can obtain the joint distribution of the whole aggregate network with all links

$$P(A^{\mathcal{O}}|\theta) = \prod_{i<j} \left[\binom{L}{A_{ij}^{\mathcal{O}}} \cdot \theta^{A_{ij}^{\mathcal{O}}} (1-\theta)^{L-A_{ij}^{\mathcal{O}}} \right].$$

The logical aggregate mechanism "AND" is also common in real life, and we denote the mapping with logical relationship "AND" by φ_{AND}. Thus, we have

$$A^{\mathcal{O}} = \varphi_{\text{AND}}(\mathbf{M}) = \prod_{\alpha=1}^{L} M^\alpha,$$

and its elements can be specified by

$$A_{ij}^{\mathcal{O}} = \varphi_{\text{AND}}(\mathbf{M}_{ij}) = \prod_{\alpha=1}^{L} M_{ij}^\alpha.$$

The "AND" aggregation will also map an unweighted multiplex network to an unweighted monoplex network. For example, assuming that the α-th layer M^α is an undirected, unweighted network generated by the ER network model with parameter θ^α, the distribution of an individual link $A_{ij}^{\mathcal{O}}$ in the aggregate network $A^{\mathcal{O}}$ with "AND" is submitted to a Bernoulli distribution

$$P(A_{ij}^{\mathcal{O}} = k) = \begin{cases} \prod_\alpha \theta^\alpha, & \text{if } k = 1 \\ 1 - \prod_\alpha \theta^\alpha, & \text{if } k = 0 \end{cases}.$$

Then, we have the joint distribution of the aggregate network

$$P(A^{\mathcal{O}}|\Theta) = \prod_{i<j} \left[\prod_\alpha \theta^\alpha \right]^{A_{ij}^{\mathcal{O}}} \cdot \left[1 - \prod_\alpha \theta^\alpha \right]^{1-A_{ij}^{\mathcal{O}}}.$$

Supplementary Note 2: Complete algorithms

In this section we will present complete algorithms for the specific cases we considered in the Results section. Notice that we have employed the configuration model for illustration, we will introduce this network model briefly. Configuration model is a kind of generative model [15,16], which can generate a network by a vector $\vec{d}$ that encodes the degree sequence, where the i-th component $d(i)$ indicates the degree of node i. Specifically, the degrees of the vertices are represented by half-links, and can be regarded as stubs. In undirected networks, the sum of all stubs must be even, and it equals the twice of the number of edges in the network, i.e., $||\vec{d}||_1 = \sum_i d(i) = 2m$, where $||\cdot||_1$ indicates the L^1-norm of a vector, and m represents the number of edges. Given a degree sequence $\vec{d}$, we can calculate the edge probability between any two nodes i and j when generating a network. A stub of node i can be connected to $2m-1$ other stubs (there are $2m$ stubs altogether, and we have to exclude the one we are currently considering). Notice that node j has $d(j)$ stubs among all stubs, and the stub of node i can be connected to these $d(j)$ stubs with the same probability (because of the uniform distribution). Thus, the probability of a stub of node i being connected to one of these $d(j)$ stubs is $d(j)/(2m-1)$. Since node i has $d(i)$ stubs, the probability of i being connected to j is $(d(i)\cdot d(j))/(2m-1)$, i.e., $(d(i)\cdot d(j))/(||\vec{d}||_1 - 1)$. Thus, whether an edge exists is submitted to a Bernoulli distribution with $p_{ij} = (d(i)\cdot d(j))/(||\vec{d}||_1 - 1)$, when generating a network by the configuration model. In the multiplex network case, the edge probability between two nodes i and j in layer α, denoted by p_{ij}^{α}, is determined by the degree sequence in layer α, i.e., $\vec{d^{\alpha}}$. Similarly, the edge probability is submitted to a Bernoulli distribution with

$$p_{ij}^{\alpha} = \frac{d^{\alpha}(i)\cdot d^{\alpha}(j)}{||d^{\alpha}||_1 - 1}. \tag{1}$$

Then we can write the likelihood function

$$P(A^{\mathcal{O}}, \Gamma | \vec{d^1}, \vec{d^2}) = \sum_{M^1, M^2} P(A^{\mathcal{O}}, \Gamma, M^1, M^2 | \vec{d^1}, \vec{d^2}), \tag{2}$$

where vectors $\vec{d^1}$ and $\vec{d^2}$ are the degree sequences in layer 1 and layer 2, respectively. Specifically, the i-th component of the vector $\vec{d^1}$ indicates the degree of node i in layer 1, i.e., $d^1(i)$. By employing the Jensen's inequality, we thus have

$$\ln P(A^{\mathcal{O}}, \Gamma | \vec{d^1}, \vec{d^2}) \geq \sum_{M^1, M^2} Q(M^1, M^2) \ln \frac{P(A^{\mathcal{O}}, \Gamma, M^1, M^2 | \vec{d^1}, \vec{d^2})}{Q(M^1, M^2)}. \tag{3}$$

Notice that in the Jensen's inequality Eq. (3), the equality holds if and only if

$$\begin{aligned} Q(M^1, M^2) &= \frac{P(A^{\mathcal{O}}, \Gamma, M^1, M^2 | \vec{d^1}, \vec{d^2})}{\sum\limits_{M^1, M^2} P(A^{\mathcal{O}}, \Gamma, M^1, M^2 | \vec{d^1}, \vec{d^2})} \\ &= \frac{P(A^{\mathcal{O}}, \Gamma, M^1, M^2 | \vec{d^1}, \vec{d^2})}{P(A^{\mathcal{O}}, \Gamma | \vec{d^1}, \vec{d^2})} \\ &= P(M^1, M^2 | A^{\mathcal{O}}, \Gamma, \vec{d^1}, \vec{d^2}). \end{aligned} \tag{4}$$

For simplicity, we denote

$$J(Q, D) = \sum_{M^1, M^2} Q(M^1, M^2) \ln \frac{P(A^{\mathcal{O}}, \Gamma, M^1, M^2 | \vec{d^1}, \vec{d^2})}{Q(M^1, M^2)}, \tag{5}$$

indicating J is a function of distribution Q and parameters D (i.e., $\vec{d^1}$ and $\vec{d^2}$). Thus, in the E-step, we maximize the function $J(Q, D)$ with respect to the distribution Q while keeping $\vec{d^1}$ and $\vec{d^2}$ constants, i.e.,

$$\begin{aligned} & Q(M^1, M^2) \\ =& \frac{P(A^{\mathcal{O}}, \Gamma, M^1, M^2 | \vec{d^1}, \vec{d^2})}{\sum\limits_{T^1, T^2} P(A^{\mathcal{O}}, \Gamma, T^1, T^2 | \vec{d^1}, \vec{d^2})} \\ =& \frac{\mathbb{1}_{\{\varphi(M^1, M^2) = A^{\mathcal{O}}\}} \cdot \mathbb{1}_{\{\Gamma \in \mathbf{M}\}} \cdot \prod\limits_{i<j} \prod\limits_{\alpha=1}^{2} (p_{ij}^{\alpha})^{M_{ij}^{\alpha}} \cdot (1 - p_{ij}^{\alpha})^{1 - M_{ij}^{\alpha}}}{\sum\limits_{T^1, T^2} \mathbb{1}_{\{\varphi(T^1, T^2) = A^{\mathcal{O}}\}} \cdot \mathbb{1}_{\{\Gamma \in \mathbf{T}\}} \cdot \prod\limits_{i<j} \prod\limits_{\alpha=1}^{2} (p_{ij}^{\alpha})^{T_{ij}^{\alpha}} \cdot (1 - p_{ij}^{\alpha})^{1 - T_{ij}^{\alpha}}} \end{aligned} \tag{6}$$

Notice that the terms $\mathbb{1}_{\{\varphi(M^1, M^2) = A^{\mathcal{O}}\}}$ and $\mathbb{1}_{\{\Gamma \in \mathbf{M}\}}$ are indicative functions. The indicative function $\mathbb{1}_{\{}1\}$ equals to 1 if the condition in brace is satisfied, and equals to 0 otherwise. Thus, the term $\mathbb{1}_{\{\varphi(M^1, M^2) = A^{\mathcal{O}}\}}$ describes the constraint if the multiplex structure M^1 and M^2 generates the aggregate network $A^{\mathcal{O}}$, and the term $\mathbb{1}_{\{\Gamma \in \mathbf{M}\}}$ indicates the constraint if M satisfies the partial observations Γ. The term $\prod_{i<j} \prod_{\alpha=1}^{2} (p_{ij}^{\alpha})^{M_{ij}^{\alpha}} \cdot (1 - p_{ij}^{\alpha})^{1 - M_{ij}^{\alpha}}$ indicates the likelihood of a given structure of $\mathbf{M}$, when denoting the edge probability between nodes i and j in layer α by p_{ij}^{α}. Obviously, the denominator is the summation over all possible multiplex networks $\mathbf{M}$ that satisfy the constraints. Notice that the term p_{ij}^{α} describes the link probability between nodes i and j in layer α, and we employed the configuration model for illustration in this article, i.e.,

$$p_{ij}^{\alpha} = \frac{d^{\alpha}(i) \cdot d^{\alpha}(j)}{||\vec{d^{\alpha}}||_1 - 1}. \tag{7}$$

Thus, the explicit functional form of $Q(\mathbf{M})$ becomes

$$
\begin{aligned}
&Q(\mathbf{M})\\
=&\frac{\mathbb{1}_{\{\varphi(M^1,M^2)=A^{\mathcal{O}}\}}\cdot\mathbb{1}_{\{\Gamma\in\mathbf{M}\}}\cdot\prod_{i<j}\prod_{\alpha=1}^{2}[\frac{d^\alpha(i)\cdot d^\alpha(j)}{||\vec{d^\alpha}||_1-1}]^{M^\alpha_{ij}}\cdot[1-\frac{d^\alpha(i)\cdot d^\alpha(j)}{||\vec{d^\alpha}||_1-1}]^{1-M^\alpha_{ij}}}{\sum_{T^1,T^2}\mathbb{1}_{\{\varphi(T^1,T^2)=A^{\mathcal{O}}\}}\cdot\mathbb{1}_{\{\Gamma\in\mathbf{T}\}}\cdot\prod_{i<j}\prod_{\alpha=1}^{2}[\frac{d^\alpha(i)\cdot d^\alpha(j)}{||\vec{d^\alpha}||_1-1}]^{T^\alpha_{ij}}\cdot[1-\frac{d^\alpha(i)\cdot d^\alpha(j)}{||\vec{d^\alpha}||_1-1}]^{1-T^\alpha_{ij}}}\\
=&\prod_{i<j}\frac{\mathbb{1}_{\{\varphi(M^1_{ij},M^2_{ij})=A^{\mathcal{O}}_{ij}\}}\cdot\mathbb{1}_{\{\Gamma\in\mathbf{T}\}}\cdot\prod_{\alpha=1}^{2}[\frac{d^\alpha(i)\cdot d^\alpha(j)}{||\vec{d^\alpha}||_1-1}]^{M^\alpha_{ij}}\cdot[1-\frac{d^\alpha(i)\cdot d^\alpha(j)}{||\vec{d^\alpha}||_1-1}]^{1-M^\alpha_{ij}}}{\sum_{T^1_{ij},T^2_{ij}}\mathbb{1}_{\{\varphi(T^1_{ij},T^2_{ij})=A^{\mathcal{O}}_{ij}\}}\cdot\mathbb{1}_{\{\Gamma\in\mathbf{T}\}}\cdot\prod_{\alpha=1}^{2}[\frac{d^\alpha(i)\cdot d^\alpha(j)}{||\vec{d^\alpha}||_1-1}]^{T^\alpha_{ij}}\cdot[1-\frac{d^\alpha(i)\cdot d^\alpha(j)}{||\vec{d^\alpha}||_1-1}]^{1-T^\alpha_{ij}}}.
\end{aligned}
\tag{8}
$$

In the M-step, we differentiate Eq. (5) with respect to $\vec{d^1}$ and $\vec{d^2}$ while fixing $Q(\mathbf{M})$ as constants, and find the solution to the equations

$$
\frac{\partial}{\partial\vec{d^1},\vec{d^2}}\sum_{M^1,M^2}Q(M^1,M^2)\ln\frac{P(A^{\mathcal{O}},\Gamma,M^1,M^2|\vec{d^1},\vec{d^2})}{Q(M^1,M^2)}=0,
\tag{9}
$$

Unfortunately, the set of these equations have no close-form solutions, which we solve by approximation method. For a large network, the number m of edges is sufficiently large, and can be regarded as a constant. Then, we also regard the product $d^\alpha(i)\cdot d^\alpha(j)$ as a whole, and obtain

$$
\begin{aligned}
\frac{\partial}{\partial[d^\alpha(i)\cdot d^\alpha(j)]}\sum_{M^\alpha_{ij}=0}^{1}Q(M^\alpha_{ij})\cdot\left\{M^\alpha_{ij}\cdot\frac{\partial[d^\alpha(i)\cdot d^\alpha(j)]}{d^\alpha(i)\cdot d^\alpha(j)}-(1-M^\alpha_{ij})\cdot\frac{\partial[d^\alpha(i)\cdot d^\alpha(j)]}{2m-1-d^\alpha(i)\cdot d^\alpha(j)}\right\}=0,\\
\forall i=1,2,\cdots,N,j=1,2,\cdots,N,\alpha=1,2,\cdots,L.
\end{aligned}
\tag{10}
$$

Thus,

$$
\begin{aligned}
\sum_{M^\alpha_{ij}=0}^{1}Q(M^\alpha_{ij})\cdot\left[\frac{M^\alpha_{ij}}{d^\alpha(i)\cdot d^\alpha(j)}-\frac{1-M^\alpha_{ij}}{2m-1-d^\alpha(i)\cdot d^\alpha(j)}\right]=0,\\
\forall i=1,2,\cdots,N,j=1,2,\cdots,N,\alpha=1,2,\cdots,L.
\end{aligned}
\tag{11}
$$

After simplification, we have

$$
d^\alpha(i)\cdot d^\alpha(j)=(2m-1)\cdot Q(M^\alpha_{ij})\cdot M^\alpha_{ij},\forall i,j,\alpha.
\tag{12}
$$

Then, we sum over all node j, obtaining

$$\sum_{j\neq i} d^{\alpha}(i)\cdot d^{\alpha}(j)=\sum_{j\neq i}(2m-1)\cdot Q(M_{ij}^{\alpha})\cdot M_{ij}^{\alpha},\ \forall i,\alpha, \tag{13}$$

and

$$d^{\alpha}(i)=\frac{2m-1}{2m-d^{\alpha}(i)}\sum_{j\neq i} Q(M_{ij}^{\alpha})\cdot M_{ij}^{\alpha},\ \forall i,\alpha. \tag{14}$$

Since we have assumed that m is sufficiently large, we finally obtain

$$d^{\alpha}(i)=\sum_{j\neq i} Q(M_{ij}^{\alpha})\cdot M_{ij}^{\alpha}=\sum_{j\neq i} E(M_{ij}^{\alpha}),\ \forall i,\alpha. \tag{15}$$

Next we will briefly perform the complexity analysis. According to Eq. (6), we have the posterior

$$Q(M^1,M^2)=\frac{\mathbb{1}_{\{\varphi(M^1,M^2)=A^{\mathcal{O}}\}}\cdot\mathbb{1}_{\{\Gamma\in\mathbf{M}\}}\cdot\prod_{i<j}\prod_{\alpha=1}^{2}(p_{ij}^{\alpha})^{M_{ij}^{\alpha}}\cdot(1-p_{ij}^{\alpha})^{1-M_{ij}^{\alpha}}}{\sum_{T^1,T^2}\mathbb{1}_{\{\varphi(T^1,T^2)=A^{\mathcal{O}}\}}\cdot\mathbb{1}_{\{\Gamma\in\mathbf{T}\}}\cdot\prod_{i<j}\prod_{\alpha=1}^{2}(p_{ij}^{\alpha})^{T_{ij}^{\alpha}}\cdot(1-p_{ij}^{\alpha})^{1-T_{ij}^{\alpha}}} \tag{16}$$

where the term $\mathbb{1}_{\{\varphi(M^1,M^2)=A^{\mathcal{O}}\}}$ and $\mathbb{1}_{\{\Gamma\in\mathbf{M}\}}$ are indicative functions: $\mathbb{1}_{\{\cdot\}}$ equals to 1 if the condition in the brace is satisfied, and 0 otherwise. Thus, the term $\mathbb{1}_{\{\varphi(M^1,M^2)=A^{\mathcal{O}}\}}$ describes if the multiplex structure $\mathbf{M}$ generates the aggregate network $A^{\mathcal{O}}$, and the term $\mathbb{1}_{\{\Gamma\in\mathbf{M}\}}$ indicates if $\mathbf{M}$ satisfies the partial observations Γ. The term $\prod_{i<j}\prod_{\alpha=1}^{2}(p_{ij}^{\alpha})^{M_{ij}^{\alpha}}\cdot(1-p_{ij}^{\alpha})^{1-M_{ij}^{\alpha}}$ indicates the likelihood of a given structure of $\mathbf{M}$, where the edge probability between nodes i and j in layer α is denoted by p_{ij}^{α}. Notice that the denominator is the summation over all possible multiplex networks $\mathbf{M}$ that satisfy the constraints. In fact, there are too many potential multiplex structure $\mathbf{M}$ that satisfy the constraints. The computational complexity $O(L\cdot m\cdot(2^L-1)^m)$ is an exponential function of the number of observed edges m, since there are $(2^L-1)^m$ possible items in the event space (Supplementary Figure 3a), where L is the number of layers. Assuming the independence of each link (and in each layer), we can extract the product operator,

$$Q(M^1,M^2)=\prod_{i<j}\frac{\mathbb{1}_{\{\varphi(M_{ij}^1,M_{ij}^2)=A_{ij}^{\mathcal{O}}\}}\cdot\mathbb{1}_{\{\Gamma_{ij}^{\alpha}\in\mathbf{M}_{ij}^{\alpha}\}}\cdot\prod_{\alpha=1}^{2}(p_{ij}^{\alpha})^{M_{ij}^{\alpha}}\cdot(1-p_{ij}^{\alpha})^{1-M_{ij}^{\alpha}}}{\sum_{T_{ij}^1,T_{ij}^2}\mathbb{1}_{\{\varphi(T_{ij}^1,T_{ij}^2)=A^{\mathcal{O}}\}}\cdot\mathbb{1}_{\{\Gamma_{ij}^{\alpha}\in\mathbf{T}_{ij}^{\alpha}\}}\cdot\prod_{\alpha=1}^{2}(p_{ij}^{\alpha})^{T_{ij}^{\alpha}}\cdot(1-p_{ij}^{\alpha})^{1-T_{ij}^{\alpha}}}. \tag{17}$$

Therefore, the probability space can be presented as the product of m probability spaces, where there are totally $(2^L - 1)$ possible items in each event space (Supplementary Figure 3b), indicating $m \cdot (2^L - 1)$ parameters. Since the computational complexity per iteration scales as $O(L \cdot m^2 \cdot (2^L - 1))$ (L is a constant), the computational complexity drops from exponential to polynomial in the computation of $Q(\mathbf{M})$. Although there are various $\mathbf{M}$ that can generate the same aggregate topology $A^{\mathcal{O}}$, it is not necessary to select all possible $\mathbf{M}$ satisfying the constraints, and calculate the corresponding probabilities (which has an exponential growth). Instead, we separate the potential event space of $A^{\mathcal{O}}$ into the product of event spaces of every link of $A^{\mathcal{O}}$, which drops the computational complexity from exponential to polynomial as we mentioned above.

The configuration model is a relative general and common model that allows an arbitrary degree sequence, while Erdos-Rényi random network model may lose such generality. Further, some other models including latent variable models may suffer complicated derivation. Therefore, we choose the configuration model for illustration in this article. There is no doubt that each generative model has its unique merit, and every analytical model can be included in our proposed framework. For example, the Erdos-Rényi random network model generates a network with a single parameter θ, describing the link probability between any two nodes. Then, the explicit functional form of $Q(\mathbf{M})$ in our framework becomes

$$Q(M^1, M^2) = \frac{\mathbb{1}_{\{\varphi(M^1,M^2)=A^{\mathcal{O}}\}} \cdot \mathbb{1}_{\{\Gamma \in \mathbf{M}\}} \cdot \prod_{i<j} \prod_{\alpha=1}^{2} (\theta^\alpha)^{M_{ij}^\alpha} \cdot (1-\theta^\alpha)^{1-M_{ij}^\alpha}}{\sum_{T^1,T^2} \mathbb{1}_{\{\varphi(T^1,T^2)=A^{\mathcal{O}}\}} \cdot \mathbb{1}_{\{\Gamma \in \mathbf{T}\}} \cdot \prod_{i<j} \prod_{\alpha=1}^{2} (\theta^\alpha)^{T_{ij}^\alpha} \cdot (1-\theta^\alpha)^{1-T_{ij}^\alpha}}, \tag{18}$$

where θ^α corresponds to the link probability in layer α.

In addition, as for the data other than node-pair interactions that may also be helpful, we emphasize that such information can be incorporated into our proposed method. Generally, if data D is available, we can calculate the posterior probability $P(\mathbf{M}|A^{\mathcal{O}}, D, \theta)$ instead of $P(\mathbf{M}|A^{\mathcal{O}}, \Gamma, \theta)$. For example, we can employ the stochastic block model instead configuration model if we know the group of nodes. Denote the group that contains node i by δ_i, stochastic matrices by $W^\alpha \in \mathbf{R}^{K \times K}$,$\alpha = 1, 2, \cdots, K$ (K is the number of groups). Then, the explicit functional form of $P(\mathbf{M}|A^{\mathcal{O}}, D, \theta)$ becomes,

$$Q(M^1, M^2) = \frac{\mathbb{1}_{\{\varphi(M^1,M^2)=A^{\mathcal{O}}\}} \cdot \mathbb{1}_{\{\Gamma \in \mathbf{M}\}} \cdot \prod_{i<j} \prod_{\alpha=1}^{2} (w_{\delta_i,\delta_j}^\alpha)^{M_{ij}^\alpha} \cdot (1-w_{\delta_i,\delta_j}^\alpha)^{1-M_{ij}^\alpha}}{\sum_{T^1,T^2} \mathbb{1}_{\{\varphi(T^1,T^2)=A^{\mathcal{O}}\}} \cdot \mathbb{1}_{\{\Gamma \in \mathbf{T}\}} \cdot \prod_{i<j} \prod_{\alpha=1}^{2} (w_{\delta_i,\delta_j}^\alpha)^{T_{ij}^\alpha} \cdot (1-w_{\delta_i,\delta_j}^\alpha)^{1-T_{ij}^\alpha}}, \tag{19}$$

where $w_{g,h}^{\alpha}$ represents the link probability between any two nodes in layer α from group g and h, respectively. Similarly, we can leverage any other available information within the Bayesian posterior estimation framework.

Supplementary Note 3: Estimation theory

Specifically, supposing that θ is a parameter (scalar) to be estimated from random variable x submitted to the probability density function $f(x;\theta)$, the variance of any unbiased estimator $\hat{\theta}$ is bounded by the inverse of the Fisher information $I(\theta)$, which is defined by

$$I(\theta) = \mathbf{E}\left[\left(\frac{\partial \ln f(x;\theta)}{\partial \theta}\right)^2\right] = -\mathbf{E}\left[\frac{\partial^2 \ln f(x;\theta)}{\partial \theta^2}\right]. \tag{20}$$

Fortunately, maximum likelihood estimator performs the asymptotic normality, indicating the maximum likelihood estimate converges to a normal distribution when the sample size N approaches the infinity [17], i.e.,

$$\sqrt{N}(\hat{\theta} - \theta_0) \rightarrow G(0, I^{-1}(\theta_0)), \tag{21}$$

where θ_0 is the real value of parameter. In the proposed framework, the Fisher information matrix $\mathbf{I}(\mathbf{\Theta})$ is defined as the expectation of Hessian matrix of the logarithmic probability density function, i.e.,

$$\mathbf{I}(\mathbf{\Theta})_{i,j} = \mathbf{E}_X\left[\frac{\partial \ln f(x;\mathbf{\Theta})}{\partial \theta_i} \cdot \frac{\partial \ln f(x;\mathbf{\Theta})}{\partial \theta_j}\right] = -\mathbf{E}_X\left[\frac{\partial^2 \ln f(x;\mathbf{\Theta})}{\partial \theta_i \partial \theta_j}\right], \tag{22}$$

where x is any observation including the aggregate network $A^{\mathcal{O}}$ and partial observations Γ. According to Cramer-Rao inequality, the variance of estimator $\mathbf{D}(\hat{\theta}_i)$ satisfies

$$\mathbf{D}(\hat{\theta}_i) \geq (\mathbf{I}^{-1}(\mathbf{\Theta}))_{i,i}. \tag{23}$$

In other words, the i-th element of leading diagonal in matrix $\mathbf{I}^{-1}$ shows the lower bound of the variance of unbiased estimator $\hat{\theta}_i$. To illustrate the asymptotic behavior of variance of the estimator, we present simulations results are shown in Supplementary Figure 4, showing the variance of the proposed estimator reaches the corresponding CRLB asymptoticly.

Supplementary Note 4: Percolation process on interdependent networks

We analyze the property of robustness between the real network and the reconstructed network in this note. Recent work [18] had made pioneering contribution to calculate the size of the

giant mutual connected component μ_∞ with occupied probability p. We will follow the notations of this paper, indicating a two-layer multiplex network is composed by two networks A and B. We notice that $G_{A0}(z) = \sum_k P_A(k)z^k$, which is the generating function of the degree distribution of network A, and $G_{A1}(z) = G'_{A0}(z)/G'_{A0}(1)$. Once a fraction, $1-p$, of randomly chosen nodes are removed, the degree distribution of the remaining nodes is changed [19]. Theoretically, $\mu_\infty = xg_B(x) = yg_A(y)$, and

$$\begin{cases} x = g_A(y) \cdot p \\ y = g_B(y) \cdot p \end{cases}, \tag{24}$$

where

$$g_A(p) = 1 - G_{A0}[1 - p(1 - f_A)]. \tag{25}$$

In the equation above, f_A is a function of p that satisfies the transcendental equation $f_A = G_{A1}[1 - p(1 - f_A)]$.

Since the above process is taken place on unweighted multiplex networks, we will binary the reconstructed network by the probability distribution $Q(\mathbf{M})$. For each individual link between nodes i and j in layer α, the value Q^α_{ij} describes the probability that there exists a link by the observations. Thus, we yield an unweighted multiplex network by generating each link submitted to a Bernoulli trial for probability Q^α_{ij}. In addition, we consider the networks that all nodes belong to GMCC, which means $\mu_\infty(1) = 1$. After generating the network by Bernoulli trials for each link, we add links by the rank of link reliability until all nodes are in the giant mutual connected component. We consider the function $\mu_\infty(p)$ to analyze the robustness of the real multiplex network and reconstructed networks.

Supplementary Note 5: Random walk process in interconnected multiplex networks

We will analyze the property of navigability between the real network and the reconstructed network in this note. Recent work [6] has made pioneering contribution to analyze the random walk process in an interconnected multiplex network. A walker walks along a general network by four types of parameters, which are i) $\mathcal{P}^{\alpha\alpha}_{ii}$, the probability for staying at the same node i and in the same layer α; ii) $\mathcal{P}^{\alpha\alpha}_{ij}$, the probability for walking from node i to its neighbor node j in the same layer α; iii) $\mathcal{P}^{\alpha\beta}_{ii}$, the probability for switching from layer α to layer β while staying at the same node i; iv) $\mathcal{P}^{\alpha\beta}_{ij}$, the probability for walking from node i in layer α to node j in layer β. For an interconnected multiplex network such as a multiplex transportation network, $\mathcal{P}^{\alpha\alpha}_{ii}$ indicates the probability that a person stays put without going anywhere, and $\mathcal{P}^{\alpha\beta}_{ii}$ indicates that a person switches the means

of transportation but still stays at the same region. Similarly, $\mathcal{P}_{ij}^{\alpha\alpha}$ indicates the probability that a person goes to node j from node i without changing means of transportation, while $\mathcal{P}_{ij}^{\alpha\beta}$ equals to 0.

Thus, the probability for finding the walker at node j in layer β at time $t + \Delta t$ is given by

$$p_{j\beta}(t+\Delta t) = \mathcal{P}_{jj}^{\alpha\alpha} \cdot p_{j\beta}(t) + \sum_{\alpha \neq \beta} \mathcal{P}_{jj}^{\alpha\beta} \cdot p_{j\alpha}(t) + \sum_{i \neq j} \mathcal{P}_{ij}^{\beta\beta} \cdot p_{i\alpha}(t). \quad (26)$$

Practically, we introduce a real number $p_{inter}, 0 < p_{inter} < 1$ to indicate the probability for a person to change vehicle. Thus, given a multiplex network $\mathbf{M}$, we have

$$\mathcal{P}_{ii}^{\alpha\beta} = \frac{p_{inter}}{L-1} \; (\forall i, \forall \alpha \neq \beta), \quad (27)$$

$$\mathcal{P}_{ij}^{\alpha\alpha} = \begin{cases} (1-p_{inter}) \cdot \frac{M_{ij}^{\alpha}}{k_i^{\alpha}}, \text{if } \; k_i^{\alpha} \neq 0 \\ 0, \text{if } \; k_i^{\alpha} = 0 \end{cases} \quad (\forall \alpha, \forall i \neq j), \quad (28)$$

and

$$\mathcal{P}_{ii}^{\alpha\alpha} = \begin{cases} 1-p_{inter}, \text{if } \; k_i^{\alpha} = 0 \\ 0, \text{if } \; k_i^{\alpha} \neq 0 \end{cases} \quad (\forall \alpha, \forall i). \quad (29)$$

The process described in Eq. (26) is a Markov process, since

$$\sum_{\beta} \sum_{j} \mathcal{P}_{ij}^{\alpha\beta} = 1, \forall \alpha, i, \quad (30)$$

We mainly study the navigability of the interconnected multiplex network by the coverage $\phi(t)$, which is the regions been visited until time t by the walkers from a random chosen node. Note that the region been visited indicates that the node is visited despite of layers. For example, the region i is visited if node i in any layer is visited, because the node i in each layer indicates the same region.

Supplementary Note 6: Spreading process in temporal networks

We first introduce epidemic spreading process in a single-layer network. There are many models describing epidemic spreading process, such as the susceptible-infected (SI) model, the susceptible-infected-susceptible (SIS) model and the susceptible-infected-recovered (SIR) model [20]. Here we take the SI model as an illustration. In a temporal network, the topology may change at each time t. Thus, we employ a multiplex network $\mathbf{M}$ to describe the time-varying topology,

where layer M^α ($\alpha = 1, 2, \cdots T$) indicates the adjacency matrix at time $t = \alpha$. Then, we study the spreading of an epidemic disease with SI model in such a multiplex network, indicating each node i of the network has only two states, "susceptible" or "infected" ($z_i = S$ or $z_i = I$).

A susceptible node is a temporarily healthy node, which can be infected by any infected neighbor node. Once node i is infected at time $t = \beta$, its state changes to "susceptible" since time $t = \beta$. At each step, an infected node will infect the susceptible nodes connected to it with probability λ, which is called the infectious rate. Since link reliability Q_{ij}^α describes the probability that there is a link from node i to j, the probability for node j being infected by node i at time $t = \alpha$ will be $\mathbf{1}_{\{z_i = I\}} \cdot \lambda \cdot Q_{ij}^\alpha$. Then, the probability for a susceptible node j being infected at time $t = \alpha$ is $1 - \prod_i (1 - \mathbf{1}_{\{z_i = I\}} \cdot \lambda \cdot Q_{ij}^\alpha)$. We consider the proportion of infected nodes $I(T)$ at time $t = T$, i.e., $I(T) = \sum_i \mathbf{1}_{\{z_i = I\}}/N$ to study the spreading process in such multiplex networks.

Supplementary Note 7: Approximations of parameters

We would provide more detailed analyses for the approximations of $\hat{r}$ and $\hat{v}$ here. We first present the approximated ratio of average degree $\hat{r}$ of two layers ranging c from 0 to 1 for nine real-world networks, where the fraction of partial observations c indicates the ratio between the number of observed edges in layers and that of all edges in the multiplex network. The nine curves show different patterns, and there is not a universal approximation for all empirical networks (Supplementary Figure 14a). In the article, we take the approximation $\hat{r} \approx r^c$ for all considered networks in empirical calculations, since $\hat{r}(r, 0) = 1$ and $\hat{r}(r, 1) = r$. The Pearson coefficient between them reaches 0.89, indicating the approximation is valid for common empirical networks to a certain degree (Supplementary Figure 14b).

Further, even though we have an approximation with higher estimation precision, it may not contribute to the analysis for the reconstructability distinctly. For example, we performed a detail analysis for an individual network (Air Transp. U.S.) (Supplementary Figure 14c), where the approximation $\hat{r} \approx r^c$ reaches 0.79 in this network (Supplementary Figure 14d). Further, we designed a new approximation with higher precision of $\hat{r}$ by $\hat{r} \approx 1 + (r \cdot c^{\frac{1}{4}} - 1) \cdot c^{\frac{1}{4}}$ for this specific network. By employed the new approximation, we have a higher correlation with a Pearson coefficient of 0.98 (Supplementary Figure 14e). However, we did not recommend the new approximation ($\hat{r} \approx 1 + (r \cdot c^{\frac{1}{4}} - 1) \cdot c^{\frac{1}{4}}$), although it performs well on the estimation of $\hat{r}$. There are two reasons. First, the approximation with higher precision will not reach a better performance in the final results, i.e., the relationship between accuracy of reconstruction and the discrimination

indicator for these two different approximations are the same, and the Pearson correlations are both 0.97 (Supplementary Figure 14f). Second, the approximation with higher precision has a precise estimation only in the air transportation multiplex network (U.S.), and it is difficult to be applied to other cases in practice because of the complex form.

For $\hat{v}$, we approximate it by $\hat{v} \approx c \cdot v$ since $\hat{v}(v, 0) = 0$ and $\hat{v}(v, 1) = v$, indicating a linear form. This approximation reaches a Pearson correlation of 0.77 for the nine considered networks (Supplementary Figure 15a), and the final results show the linear relationship between accuracy of reconstruction and the discrimination indicator, where the Pearson correlation reaches 0.96 (Supplementary Figure 15b). Moreover, we also designed a nonlinear approximation with a higher precision for $\hat{v}$ by $\hat{v} \approx c \cdot v^{\frac{1}{10}}$, reaching a higher correlation for all considered networks (Pearson coefficient 0.94) (Supplementary Figure 15c). However, this nonlinear approximation only made a slightly improvement with the final result, increasing the Pearson correlation between accuracy of reconstruction and the discrimination indicator from 0.96 to 0.97 (Supplementary Figure 15d).

As we analyzed above, although several empirical networks may not agree with the approximations with the simplified form, it provided a convenient way for calculation in practice. Moreover, approximations that are more precise but more complex would only slightly improve the final results. Therefore, we could draw the conclusion that although several simplified approximations are made in empirical calculations, our main results and conclusions are untouched.

Supplementary References

1. Chicco, D. & Jurman, G. The advantages of the matthews correlation coefficient (mcc) over f1 score and accuracy in binary classification evaluation. *BMC genomics* **21**, 1–13 (2020).

2. De Bacco, C., Power, E. A., Larremore, D. B. & Moore, C. Community detection, link prediction, and layer interdependence in multilayer networks. *Physical Review E* **95**, 042317 (2017).

3. Tarrés-Deulofeu, M., Godoy-Lorite, A., Guimera, R. & Sales-Pardo, M. Tensorial and bipartite block models for link prediction in layered networks and temporal networks. *Physical Review E* **99**, 032307 (2019).

4. Chen, B. L., Hall, D. H. & Chklovskii, D. B. Wiring optimization can relate neuronal structure and function. *Proceedings of the National Academy of Sciences* **103**, 4723–4728 (2006).

5. De Domenico, M., Porter, M. A. & Arenas, A. Muxviz: a tool for multilayer analysis and visualization of networks. *Journal of Complex Networks* **3**, 159–176 (2015).

6. De Domenico, M., Solé-Ribalta, A., Gómez, S. & Arenas, A. Navigability of interconnected networks under random failures. *Proc Natl Acad Sci USA* **111**, 8351–8356 (2014).

7. G'enois, M. & Barrat, A. Can co-location be used as a proxy for face-to-face contacts? *EPJ Data Science* **7**, 11 (2018).

8. Stark, C. *et al.* Biogrid: a general repository for interaction datasets. *Nucleic acids research* **34**, D535–D539 (2006).

9. De Domenico, M., Nicosia, V., Arenas, A. & Latora, V. Structural reducibility of multilayer networks. *Nature communications* **6**, 6864 (2015).

10. Costanzo, M. *et al.* The genetic landscape of a cell. *science* **327**, 425–431 (2010).

11. Cardillo, A. *et al.* Emergence of network features from multiplexity. *Sci Rep* **3**, 1344 (2013).

12. Paranjape, A., Benson, A. R. & Leskovec, J. Motifs in temporal networks. In *Proceedings of the Tenth ACM International Conference on Web Search and Data Mining*, 601–610 (ACM, 2017).

13. Leskovec, J., Huttenlocher, D. & Kleinberg, J. Governance in social media: A case study of the wikipedia promotion process. In *Fourth International AAAI Conference on Weblogs and Social Media* (2010).

14. Panzarasa, P., Opsahl, T. & Carley, K. M. Patterns and dynamics of users' behavior and interaction: Network analysis of an online community. *Journal of the American Society for Information Science and Technology* **60**, 911–932 (2009).

15. Newman, M. E. The structure and function of complex networks. *SIAM Rev* **45**, 167–256 (2003).

16. Newman, M. *Networks* (Oxford university press, 2018).

17. Newey, W. K. & McFadden, D. Large sample estimation and hypothesis testing. *Handbook of econometrics* **4**, 2111–2245 (1994).

18. Buldyrev, S. V., Parshani, R., Paul, G., Stanley, H. E. & Havlin, S. Catastrophic cascade of failures in interdependent networks. *Nature* **464**, 1025–1028 (2010).

19. Newman, M. E. Spread of epidemic disease on networks. *Physical review E* **66**, 016128 (2002).

20. Bailey, N. T. *et al. The mathematical theory of infectious diseases and its applications* (Charles Griffin & Company Ltd, 5a Crendon Street, High Wycombe, Bucks HP13 6LE., 1975).
